# Spatial Lymphocyte Dynamics in Lymph Nodes Predicts the Cytotoxic T Cell Frequency Needed for HIV Infection Control

**DOI:** 10.3389/fimmu.2019.01213

**Published:** 2019-06-11

**Authors:** Dmitry Grebennikov, Anass Bouchnita, Vitaly Volpert, Nikolay Bessonov, Andreas Meyerhans, Gennady Bocharov

**Affiliations:** ^1^Moscow Institute of Physics and Technology, National Research University, Dolgoprudny, Russia; ^2^Marchuk Institute of Numerical Mathematics, Russian Academy of Sciences, Moscow, Russia; ^3^Peoples' Friendship University of Russia (RUDN University), Moscow, Russia; ^4^Division of Scientific Computing, Department of Information Technology, Uppsala University, Uppsala, Sweden; ^5^Institut Camille Jordan, UMR 5208 CNRS, University Lyon 1, Villeurbanne, France; ^6^INRIA Team Dracula, INRIA Lyon La Doua, Villeurbanne, France; ^7^Institute of Problems of Mechanical Engineering, Russian Academy of Sciences, Saint Petersburg, Russia; ^8^Infection Biology Laboratory, Department of Experimental and Health Sciences, Universitat Pompeu Fabra, Barcelona, Spain; ^9^Institució Catalana de Recerca i Estudis Avançats (ICREA), Barcelona, Spain; ^10^Sechenov First Moscow State Medical University, Moscow, Russia

**Keywords:** lymphoid tissue, cell motility, HIV infection, cytotoxic T cell scanning, multicellular dynamics, dissipative particle dynamics, stochastic differential equation

## Abstract

The surveillance of host body tissues by immune cells is central for mediating their defense function. *In vivo* imaging technologies have been used to quantitatively characterize target cell scanning and migration of lymphocytes within lymph nodes (LNs). The translation of these quantitative insights into a predictive understanding of immune system functioning in response to various perturbations critically depends on computational tools linking the individual immune cell properties with the emergent behavior of the immune system. By choosing the Newtonian second law for the governing equations, we developed a broadly applicable mathematical model linking individual and coordinated T-cell behaviors. The spatial cell dynamics is described by a superposition of autonomous locomotion, intercellular interaction, and viscous damping processes. The model is calibrated using *in vivo* data on T-cell motility metrics in LNs such as the translational speeds, turning angle speeds, and meandering indices. The model is applied to predict the impact of T-cell motility on protection against HIV infection, i.e., to estimate the threshold frequency of HIV-specific cytotoxic T cells (CTLs) that is required to detect productively infected cells before the release of viral particles starts. With this, it provides guidance for HIV vaccine studies allowing for the migration of cells in fibrotic LNs.

## Introduction

The surveillance of host body tissues by cells of the immune system is central for mediating defense functions against invading pathogens and tumor cells (1, 2). The initial recognition of foreign antigens that leads to the induction of adaptive immune responses takes place in lymph nodes (LNs), which, by virtue of their location and structure, facilitate the interactions between immune cells (3). The motility of pathogen spread and immune cells represents relevant parameters controlling the fate of the pathogen–host interaction. *In vivo* imaging technologies have been used to quantitatively characterize target cell scanning and migration dynamics of lymphocytes within LNs (4, 5). The translation of these quantitative insights into a predictive understanding of immune system functioning in response to various perturbations critically depends on the availability of computational tools linking the individual immune cell properties with the systems response as a whole (6).

Multiscale models of the immune system provide the *in silico* tool to embed immune processes into their spatial context (7–9). A core module of the models is the mathematical framework used to describe individual cell migration in complex multicellular environments. One can distinguish two general types of modeling approaches, cellular automata-based models (CAMs), and physical models (PMs). CAMs consider a regular grid with cells that change their state in time and space according to some rules (functions of the system state). The respective computational algorithms can take the form of random walks (10) or cellular Potts models (11). Although CAMs incorporate experimentally defined characteristics of cell motion and, thus, simulate cell dynamics based on actual data, they lack quantifiable links to the underlying biophysical interactions between cells in multicellular environments and to intrinsic cell motility parameters (12). PMs of lymphocyte migration dynamics derived from the Newtonian second law offer the possibility to define cell motions in terms of the forces generated by the environment and the cell itself. Using the experimental data on cell movement, the potential functions underlying cell-to-cell interactions and intrinsic cell motility can be identified and can provide a deeper insight into the mechanical properties of cells. Thus, PMs of individual cells and coordinated cell migration represent a general and generic way to describe and predict the multicellular system dynamics for a broad range of cell numbers and external conditions (13, 14).

It is widely accepted in immunology that the physiological function of cytotoxic T cell (CTL) motility is to search for target cells, i.e., for virus-infected cells or cancer cells (15). Computational modeling studies have revealed that the search efficiency depends on the organization of the stromal environment of a tissue (16). In addition, the spatial behavior, for example, of HIV-infected target cells scanned for foreign antigens by CTLs strongly impacts the elimination efficiency of the infected targets (17, 18). Experimental investigation of live attenuated SIV vaccines clearly suggested that a robust protection against intravenous wild-type SIVmac239 challenge strongly correlates with the number and function of antigen-specific effector CTLs in LN rather than the responses of such cells in the blood (19). However, the quantitative effects of T-cell migration parameters in LNs on the efficiency of antiviral immune responses *in vivo* remain unknown.

In the current study, we have developed a physics-based description of spatial T-lymphocyte dynamics in the multicellular environment of LNs. A fundamental relationship between a cell motion and the forces acting on it is provided by Newton's second law. It is used to formulate, calibrate, and apply a generic mathematical model of coordinated T-cell migration dynamics in LNs. By choosing a first principles approach in formulating the governing equations in conjunction with published experimental data on T-cell motility in lymphoid tissues, we offer a broadly applicable generic mathematical tool linking individual and coordinated cell behaviors. The potential of the model is illustrated by an analysis of the combined effects of antigen-specific T-cell numbers and intrinsic T-cell motility parameters in LNs on the time needed to locate both mobile and non-motile HIV-infected target cells. Computed predictions of the ratio of effector CTLs to infected T cells in the LN paracortex needed for a timely detection of infected cells within 18 h postinfection, i.e., before the release of viral particles starts (20), provide a novel quantitative guide for an informed design of HIV vaccines.

## Materials and Methods

### Programming Languages and Computing Resources

All algorithms were written in C++ and compiled using G++ (version 5.4.0). Pseudorandom numbers were generated using the PCG random library (version 0.98) and the PCG64-XSL-RR algorithm (21). The seed was either specified manually (for code development) or set based on the system's random device (for computational experiments). Simulations were run on a 2-core Xeon E3-1220 v5 @3.0 GHz ×4 processor. The wxWidgets library (version 2.8.12) was used for visualization purposes. The processing of the simulation results (i.e., calculating statistical motility profiles, comparing CDFs, and plotting) was implemented in Python and R scripts.

### Model Equations of Multicellular Dynamics

According to a basic mechanics view, a system consisting of *N* cells of some mass located in a liquid milieu, interacting with each other and affected by some external field, is uniquely determined by their coordinates and velocities and is governed by the classical mechanics motion equations. In our model, each cell $i,i=\bar{1,N},$ is represented as the circle with certain mass *m*_*i*_, radius *r*_*i*_, and position of its center *x*_*i*_. The fundamental equation governing locomotion of cells is Newton's second law of motion. It can be expressed as follows:

(1) $$m_{i}{\ddot{x}}_{i}=F_{i}=\sum_{j\neq i}f_{\text{ij}}^{\text{int}}+f_{i}^{\text{mot}}+f_{i}^{\text{dis}},\;\;i=\bar{1,N},$$

where the first term on the right side specifies the net effect of the pairwise interaction forces with contacting neighbor cells, the second term stands for the cell intrinsic locomotion force, by which the cell establishes motility within the extracellular matrix (ECM) of the LN reticular network, and the last one takes into account the action of a dissipative force, taken to be proportional to the cell velocity $f_{i}^{\text{dis}}=-\mu {ẋ}_{i}$. We neglect the impact of gravity.

### Random Motility Force Sampling

The random motility force $f_{i}^{\text{mot}}$ for the *i*th cell is modeled as a stochastic vector *f*_*i*_ sampled every 30 s from certain probability distributions analogously to the inverse homogeneous correlated random walk (IHomoCRW) model (22). The motility magnitude |*f*_*i*_| = η_*i*_·*K*_*i*_ is sampled from the following Gaussian distribution: $K_{i}\in |N\left(\mu \left(K\right),\sigma^{2}\left(K\right)\right)|$. To obtain the motility magnitude |*f*_*i*_|, the sampled value *K*_*i*_ is multiplied by the arresting coefficient η_*i*_. The arresting coefficients are increased for both T cells and DCs if they establish a sufficiently long contact to temporarily arrest their inner motility as follows: (1) $\eta_{i}:=10\eta_{i}^{default}$ for T cells and DCs when the duration of an uninterrupted contact exceeded 30 s, and (2) $\eta_{i}:=100\eta_{i}^{default}$ if the contact outlasted 20 min. The cell inner motility is restored back to a default value if the contact lasted for a time longer than the sampled value *t*_*contact*_ ∈ *N*(2, 0.4) hours. The parameter η_*i*_ is also used to decrease intrinsic motility when performing *in silico* simulations to study the effect of decreased T-cell motility on target cell location efficiency (see details in Supplementary Text).

The motility direction ${\hat{f}}_{i}$ is turned from the previous direction on the angle θ_*i*_:

(2) $$\begin{array}{l} \alpha_{i}\in N\left(0,\sigma^{2}\left(\alpha \right)\right),\;\;\;\;\;\theta_{i}=\alpha_{i}\cdot \;\left(1-{\left(\frac{K_{i}}{K_{\text{max}}}\right)}^{\beta }\right),\\ \;K_{\text{max}}=\mu \left(K\right)+3\sigma \left(K\right). \end{array}$$

Here, *N*(0, σ^2^(·)) denotes a Gaussian distribution, and β is a scalar coefficient. The angle sampled from the normal distribution is multiplied by a factor depending on the sampled motility magnitude to reproduce the experimentally observed negative correlation between cell translational and turning angle speeds. Indeed, the cells do not simultaneously perform fast translational movements and large reorientations (22). Note that a similar feature was named “directional propensity” and modeled with trigonometric parameterization in a cellular Potts model to describe the motion of T cells (11). The Gaussian distribution for the motility magnitude is set so that the (μ − 3σ, μ + 3σ) range is positive. The absolute value is taken to ensure that the magnitude is non-negative. The parameter *K*_max_ provides an upper boundary for sampled values *K*_*i*_ (approximately 1 of 370 cases falls outside of the three-sigma interval). The hat above the vector denotes the normalized unit vector.

### Implementation of Contact Inhibition of Locomotion

After the stochastic vector *f*_*i*_ is sampled, it is modified in accordance with the contact inhibition of locomotion (CIL) model, as described (23). The resultant vector $f_{i}^{\text{mot}}$ is then used in the right-hand side of Equation (1). The modification consists of shifting the direction of vector *f*_*i*_ away from the neighboring cells and decreasing the magnitude of vector *f*_*i*_ proportionally to the number of neighboring cells:

(3) $$f_{i}^{\text{mot}}=\frac{\left|f_{i}\right|\;\cdot \;\left(c_{\text{inh}}{\hat{f}}_{i}+{\hat{R}}_{i}\right)}{c_{\text{inh}}+n},\;{\hat{R}}_{i}=\sum_{j,h_{\text{ij}}\leq r_{i}+r_{j}}\frac{x_{i}-x_{j}}{h_{\text{ij}}},$$

in which |*f*_*i*_| is the magnitude and ${\hat{f}}_{i}$ is the direction of the inner motility as it would be if unaffected by CIL, *n* is the number of neighboring cells in contact (such that the distance between cell centers *h*_ij_ ≤ *r*_*i*_ + *r*_*j*_), and ${\hat{R}}_{i}$ determines the net shift of the inner motility direction away from the neighboring cells, *c*_inh_is the weighting coefficient varying the level of CIL. The hat above the vector indicates that it is normalized.

### Numerical Integration of the Equations of Cell Motion

To numerically integrate the equations of motion (Equation 1), we used the first-order semi-implicit (i.e., the cell coordinate at time *t*^*n*+1^ is computed using the velocity vector $v_{i}^{n+1}$ rather than $v_{i}^{n}$) Euler method:

(4) $$v_{i}^{n+1}=\frac{m_{i}v_{i}^{n}+h\;\cdot \;\left(F_{i}^{\text{int}}\left(t^{n},x_{i}^{n}\right)+f_{i}^{\text{mot}}\left(t^{n},x_{i}^{n}\right)\right)}{m_{i}+h\;\cdot \;\mu }$$

(5) $$x_{i}^{n+1}=x_{i}^{n}+h\;\cdot \;v_{i}^{n+1}$$

in which $x_{i}^{n}$ and $v_{i}^{n}$ are the coordinate and velocity of cell *i* at the time *t*^*n*^ after *n* steps *t*^*n*^ = *t*^0^ + *h* · *n*. We note that the second-order generalization of this method, i.e., the Störmer–Verlet method, could be developed. However, it will be computationally more demanding as the cell acceleration depending on velocity due to the presence of dissipative velocity-damping viscosity forces needs to be reevaluated at each time step *t*^*n*+1^. We verified that the time step *h* = 0.02 min used in the simulations is sufficient for a stable integration of the initial value problem with the semi-implicit Euler method. To efficiently locate the neighboring cells (which is needed for intercellular force calculations and for determining the effect of CIL), we use a simple uniform-grid-based spatial neighbor search, which performs well for a densely packed multicellular environment. Note that the convergence of the integration scheme was verified by repeating simulations for a smaller time step.

### Boundary Conditions

During the model calibration process, we used periodic boundary conditions for all boundaries of a square domain. To perform *in silico* simulations in a closed ellipse-shaped domain representing a LN, we implemented a biologically based boundary condition of cell repolarization. We do not model explicitly the interaction forces between cells and the boundary (i.e., the subcapsular sinus wall). At the stage of coordinate updates (in accordance with the numerical scheme specified in the section Numerical Integration of the Equations of Cell Motion), if the proposed coordinate of cell $x_{i}^{n+1}$ is outside the boundary, the current coordinate of the cell is preserved ($x_{i}^{n+1}=x_{i}^{n}$), while the direction of the motility vector ${\vec{f}}_{i}^{\text{mot}}$ is changed to be the opposite direction of vector $v_{i}^{n+1}$, thus resulting in cell repolarization.

### Generating the Initial Spatial Configuration for Simulations Within a LN

To generate the initial spatial configuration of the immune cells within a LN, we followed the descriptions from a LN imaging study (24). The following cell subsets were considered: CD4^+^ T cells, CD8^+^ T cells, and cross-presenting migratory CD8α^int^CD103^hi^ DCs. Both T-cell subsets are distributed uniformly through the whole LN, while migratory DCs are found mainly deep in the paracortex area. To arrange cells in agreement with the experimental data, we approximated the DC-rich area as an ellipse $\Omega_{\text{DC}}^{\alpha =0.99}$. The spatial positions for DC locations are iteratively sampled from the 2D Gaussian distribution with a 99-percentile ellipse $\Omega_{\text{DC}}^{\alpha =0.99}$ and accepted if the DC with sampled coordinates lies within the LN domain Ω_LN_ and does not overlap with the other seeded DCs. After DCs are placed, the T cells are positioned uniformly through the remaining non-occupied space of Ω_LN_.

## Results

### Biophysical Parametrization of the Spatial Multicellular Dynamics

Multicellular systems dynamics can be accurately described by biophysical models as reviewed recently (14, 25). Here, we develop a physics-based mathematical model of coordinated immune cell motion that belongs to the class of self-propelled particle models (14) and, more generally, to the dissipative particle dynamics (26, 27) framework.

**Figure 1:**
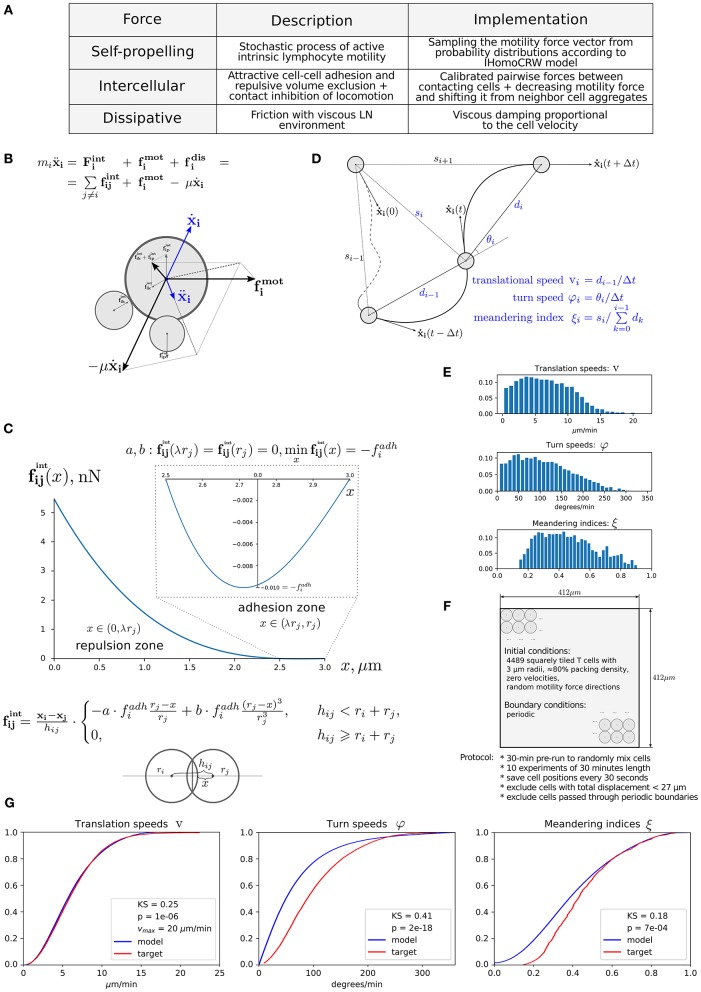
Physics-based model of multicellular system dynamics reproduces experimental data on T-cell locomotion. **(A)** The set of forces considered in the model with description of their features and implementation details. **(B)** The fundamental equation governing locomotion of cells determined by the forces exerted on cell *i*, including the repulsive–attractive interaction with neighbor cells *p* and *k*, respectively. **(C)** The parameterization of intercellular interaction force $f_{\text{ij}}^{\text{int}}$and formula definition. The calibrated force for non-specific interaction of two T cells with a radius of 3 μm is depicted. By simulation, the parameters *a* and *b* are calculated at each time step depending on the radii r_*i*_, r_*j*_ and the distances h_*ij*_, *x*, so that the condition $f_{ij}\left(\lambda r_{j}\right)=f_{ij}\left(r_{j}\right)=0,minf_{ij}\left(x\right)=-f_{i}^{adh}$ is satisfied. The parameter λ determines the relative deformation of the cells that separates the repulsive and attractive interactions between them. Parameter $f_{i}^{adh}$ represents the adhesive strength between the membranes of cells *i* and *j*. **(D)** Schematic illustration and definition of the metrics characterizing T-cell motility: translational speed, turning angle speed, and meandering index. All metrics are measured for each cell every Δ*t* seconds and pooled together to form statistical distributions. **(E)** Statistical profiles characterizing the T-cell locomotion consists of distribution histograms of translational speeds, turning angle speeds, and meandering indices. The histograms are derived from the corresponding empirical cumulative distribution functions (CDFs) available in Figure S17 from Read et al. (22), in which original *in vivo* data are presented. **(F)** The details of the 2D geometric setup for simulations used in the model calibration: spatial configuration, initial and boundary conditions, and the experimental protocol used to sample the statistical profile. **(G)** The statistical characteristics of T-cell motility coming from simulations of the calibrated model plotted against the *in vivo* histogram data (22). The statistical distributions of each metric are depicted as CDFs. The Kolmogorov–Smirnov statistics comparing the model and target CDFs are indicated with their respective *p*-values.

Immune cells in LNs are continuously interacting with each other and with stromal cells via forces of different origin, i.e., elastic (membranes), chemical (receptors), and electric. The respective forces in combination with cell intrinsic locomotion events act in concert to determine the basal intranodal motility of T cells. Figure 1A presents the overall summary of physical forces included in the model with some implementation details. The scheme of the forces exerted on cell *i* interacting with cells *p* and *k* is shown in Figure 1B. The quantitative features of the force functions are detailed in Figure 1C. Here, $f_{\text{ij}}^{\text{int}}$ is the intercellular force acting on cell *i* due to interaction with cell *j*. The pairwise cell-to-cell interactions are assumed to have a finite cutoff distance and are considered to be elastic acting along the line of cell centers. The intercellular forces $f_{\text{ij}}^{\text{int}}$ can be considered as the gradients of pairwise potentials, which are repulsive at short distances and attractive at larger distances, thus accounting for volume exclusion at the cell body and cell–cell adhesion near membranes. We consider the following cubic polynomial function to model the force exerted by cell *j* on cell *i*:

(6) $$f_{\text{ij}}^{\text{int}}=\frac{x_{i}-x_{j}}{h_{\text{ij}}}\;\cdot \;\{\begin{array}{cc} -a\;\cdot \;f^{\text{adh}}\cdot \frac{r_{j}-x}{r_{j}}+b\;\cdot \;f^{\text{adh}}\;\cdot \;{\left(\frac{r_{j}-x}{r_{j}}\right)}^{3}, & h_{\text{ij}}<r_{i}+r_{j},\\ 0, & h_{\text{ij}}\geq r_{i}+r_{j}, \end{array}$$

where *r*_*i*_ is a radius of the *i*th cell membrane, *h*_ij_ is the distance between cell centers (see Figure 1C), and *x* = *h*_ij_ − *r*_*i*_ is the distance between the center of cell *j* and the surface membrane of cell *i*. The function $a\cdot f^{\text{adh}}\cdot \frac{r_{j}-x}{r_{j}}$ describes the attraction force between two cells, and the function $b\cdot f^{\text{adh}}\cdot {\left(\frac{r_{j}-x}{r_{j}}\right)}^{3}$corresponds to a repulsive force, both calibrated as shown in Figure 1C. The coefficients *a* and *b* are set such that the minimum of function $f_{\text{ij}}^{\text{int}}$ is equal to *f*^adh^. Thus, the only remaining free parameter is *f*^adh^, the adhesive interaction strength. In the case of T cell/T cell interaction, it corresponds to weak non-specific electrical forces (electrostatic and electrodynamic) that are expected to be present between all cells according to the model of Bell (28). We calibrate this parameter by the typical value of low-adhesive forces, with which integrins present on T-cell membrane bind to their ligands present on the other cells (29). For cognate T cell/APC interactions the attraction force is much stronger as it is determined by a broad spectrum of various adhesion molecules involved in T-cell activation clusters, i.e., the immunological synapse (30). The estimated values of the intercellular interaction forces are given in Table 1. For details on the data-based T-cell motility model calibration, see Supplementary Text.

**Table 1:** Set of calibrated model parameters used as a baseline for all simulations.

**Parameter**	**Description**	**Value**	**References**
*m*_*TC*_	T-cell mass	215 pg	(31–34)^†^^*^
*r*_*TC*_	T-cell radius	3 μm	(35)^†^
μ	Viscous damping coefficient	0.2 nN·min/μm (= 12 g/s)	(36, 37)^*^^t^
$f_{T-T}^{adh}$	Adhesive strength between T cells	0.01 nN	(28–30)^†^
λ_*TC*_	The normalized distance between the cell-to-cell interaction synapse and the cell center separating repulsive and attractive modes of T-cell interaction (model analog of nuclear-to-cytoplasmic ratio in experimental cell biology)	0.83 (with respect to *r*_TC_)	(38)^*^^t^
Δ*t*	Time step for inner motility *m*_*TC*_ update	30 s	(22) ^‡^
μ(*m*_*TC*_)	Mean of the inner motility force magnitude distribution	3 nN	(22, 39)^‡^^*^^t^
σ(*m*_*TC*_)	SD of the inner motility force magnitude distribution	0.3 nN	(22, 39) ^‡^^*^^t^
σ(α_*TC*_)	SD of the inner motility turning angle distribution	60°	(22)^‡^^t^
*c*_*inh*_	Scalar coefficient varying the level of CIL	1.0	(23)^‡^^t^
β	Scalar coefficient in parameterization of negative correlation between magnitude and turning angle of sampled inner motility	2.0	(23)^‡^^t^

† *Parameters obtained directly from experimental measurements*.

* *Parameters estimated indirectly from experimental measurements*.

‡ *Parameters derived from underlying computational models*.

t *Parameters tuned to fit cell motility profiles within the model calibration*.

The dissipative (friction) force acting on T cells describes the effect of viscous damping, which reduces the velocity of the cell. It is assumed to be proportional to the cell velocity $f_{i}^{\text{dis}}=-\mu {ẋ}_{i}$. The dissipative force acts along the line of the cell center and in opposite direction to the cell displacement. Consideration of viscous damping is appropriate for the highly viscous low-Reynolds-number environment of LNs (40). The viscous damping parameter estimate is listed in Table 1.

The random motility force $f_{i}^{\text{mot}}$ determines the traction of self-propelled lymphocytes. It represents a stochastic process of receptor-mediated cell–ECM interactions regulated by either cytoskeletal or membrane reorganizations and governed by biomechanical and intracellular molecular mechanisms (4, 13). Basically, cells establish directed caterpillar-like movement by polarizing, forming contacts between their leading edge and collagen fibers of ECM, detaching their trailing edge from ECM, and contracting. However, T lymphocytes and dendritic cells (DCs) are characterized by low-adhesive integrin interactions with the microenvironment. This allows them to adapt their direction and morphology with no need to reorganize microstructure while effectively sliding along the stromal network of fibroblastic reticular cells (41, 42). As we do not model the reticular network and the ECM microstructure in this study explicitly, this motility behavior is considered implicitly in the stochastic nature of $f_{i}^{\text{mot}}$. Note that the autonomous cell motility can also be affected by external signaling, e.g., through chemotaxis, CIL, or immunological synapse formation. The cell trajectory in the model is characterized by three quantifiable values, i.e., the translational speed, the turning angle speed, and the meandering index as explained in Figure 1D and as described in Read et al. (22). The corresponding experimental data are shown in Figure 1E. To capture the experimentally observed patterns of T-lymphocyte migration in lymphoid tissues (see Figure 1E), the T-cell motility is modeled using a random variable *f*_*i*_ with its magnitude and angle values updated every Δ*t* seconds according to the IHomoCRW recently suggested and validated (22). The IHomoCRW model was shown to reproduce the experimentally measured statistical profiles of T-cell locomotion (22). In the present model, the magnitude and direction of the random vector $f_{i}^{\text{mot}}$ are sampled from distributions provided by the experimental data (the specific rules are defined in section Materials and Methods). The key difference from the original IHomoCRW model is that it is the cell motility inducing force $f_{i}^{\text{mot}}$ rather than the cell velocity ẋ_*i*_ that is sampled and then substituted into equation (Equation 1). In addition, the random vector $f_{i}^{\text{mot}}$ can be influenced by contact effects from neighboring cells, resulting in (1) a shift of the vector $f_{i}^{\text{mot}}$ away from neighboring cells and (2) a decrease of its magnitude proportionally to their number, similar to the CIL model (23) (see details in section Materials and Methods). By default, the arresting coefficient for T cells is equal to one. For DCs, its value is estimated so that the resultant DC velocities do not exceed 5 μm/min (the estimated value is specified in Table 2).

**Table 2:** Extra parameters which are used for LN simulations.

**Parameter**	**Description**	**Value/range**	**References**
*m*_*CD*4_	CD4^+^ T-cell mass	215 ± 28 pg	(31–34)
*m*_*CD*8_	CD8^+^ T-cell mass	290 ± 28 pg	(31–34)
*m*_*DC*_	Dendritic cell mass	350 ± 28 pg	(32–34)
*r*_*DC*_	Dendritic cell radius	6.5 μm	(38, 43)
λ_*DC*_	The normalized distance between the cell-to-cell interaction synapse and the cell center separating repulsive and attractive modes of DC interaction (model analog of nuclear-to-cytoplasmic ratio in experimental cell biology)	0.5	(38)
$f_{DC}^{adh}$	Adhesive strength between specific T cells and DCs	1 nN	(28, 30, 36)
η_*DC*_	Default value for coefficient arresting inner motility of DCs	3	Tuned so that DC velocities are <5 μm/min

Overall, the mechanistic model of the spatial multicellular dynamics is formulated as a system of *N* random ordinary differential equations (44) represented by Equation (1) and embedded into the 2D geometric domain as detailed in Figure 1F. Essentially, the system is a deterministic system of ordinary differential equations on each interval of Δ*t* seconds, until the force $f_{i}^{\text{mot}}$ becomes updated. The quantitative consistency of the computational model of multicellular dynamics with experimental data on translation speed, turning angle speed, and the meandering index is illustrated in Figure 1G. The relevant components of the numerical implementation of the model (computational domain, boundary conditions, integration algorithm) are described in Materials and Methods. The dynamics of the net forces and their contributions acting on a randomly selected T cell in a simulation of multicellular dynamics are shown in Figure S1.

### Calibration of T-Cell Motility

Our model mostly operates with biophysical parameters that are either directly measurable or can be estimated indirectly such as the mass *m* (wet weight) and the radius *r* of a cell, the adhesive strength between T-cell membranes $f_{ij}^{adh}$ (measured by single cell force spectroscopy), the viscous damping coefficient μ, typical forces and velocities of T cells, and the location of demarcation between repulsive and attractive areas of a cell λ (nuclear-to-cytoplasma ratio). The other parameters that describe the random motility force or the contact inhibition of locomotion are derived using the information presented in the original IHomoCRW model (22) and the CIL model (23) with the underlying experimental data. To calibrate our model, we evaluated admissible ranges of parameters and tuned them manually to match the statistical characteristics of T-cell locomotion (22). The baseline sets of the estimated model parameters are presented in Tables 1, 2. For details of the parameter estimation, see Supplementary Text.

#### Computational Domain, Immune Cell Subsets, and Initial Configuration

**Figure 2:**
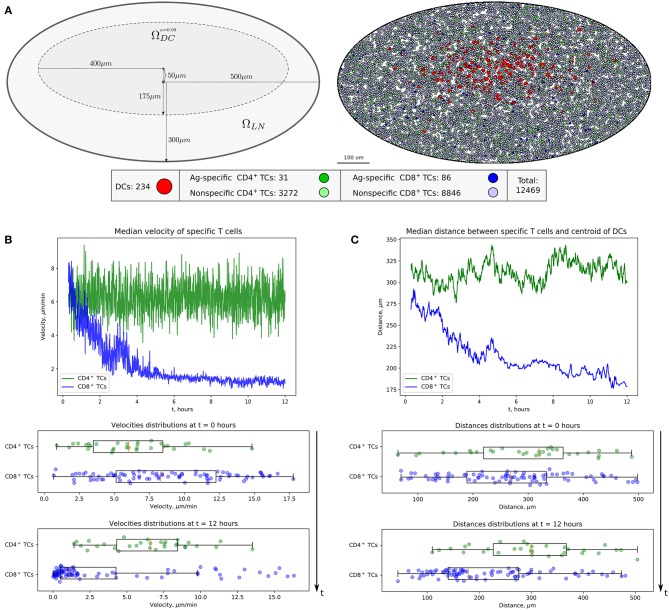
Heterogeneous dynamics of T cells in LNs. **(A)** The scheme of a LN and illustration of the initial configuration generated for simulations. DCs, CD4^+^ T cells, and CD8^+^ T cells are placed within a LN as described in the Supplementary Text with total cellularity of 12,469 cells, ≈ 80% packing density and ≈ 1% precursor frequency. **(B)** Twelve-hour kinetics of median velocities of antigen-specific CD8^+^ T and CD4^+^ T cells, and their distributions at the start and at the end of a 12-h simulation. **(C)** Twelve-hour kinetics of median distances from T cells to the centroid of DCs, measured for antigen-specific CD8^+^ T and CD4^+^ T cells, and their distributions at the start and at the end of a 12-h simulation. TC, T cell; DC, dendritic cell.

The computational domain was implemented as an ellipse-shaped 2D approximation of the bean-like cross section of a murine skin-draining LN (see Figure 2A). At the beginning, both CD4^+^ T cells (green) and CD8^+^ T cells (blue) are evenly distributed throughout the domain. Some randomly chosen T cells are considered to be antigen specific and marked in light green and blue, respectively (their numbers are specified below). The antigen-presenting cells considered in this study represent the subset of cross-presenting migratory CD8α^int^CD103^hi^ DCs, which are mainly involved in CD8^+^ T-cell immune responses and which immigrate into LNs from the periphery (24). They are normally localized in the deep parts of the T-cell zone and leave LNs slowly with a turnover rate of 6 days. For initial configuration, these DCs are spatially placed according to a Gaussian distribution with 99-percentile ellipse $\Omega_{DC}^{\alpha =0.99}$ representing the T-cell zone (see Figure 2A and section Materials and Methods).

The numbers of antigen-specific DC and T-cell subsets are estimated using published data (24), which were rescaled according to the size of the computational domain. A total population of 12,469 immune cells was considered. The total number of non-antigen-specific T cells was estimated so that about 80% of the computation domain was filled up. The precursor frequency of antigen-specific T cells, that is, their proportion in the total amount of T cells, was set to be about 1%. We consider the inflow and outflow of immune cells to the region of interest to be negligible because of the short simulation time of 12 h. The closed boundary conditions used in the simulations are specified in section Materials and Methods. The overall geometrical scheme of the computational domain and the initial configuration of the multicellular system generated for simulations are presented in Figure 2A.

#### Data Assimilation and Model Validation

To assimilate the statistical data on the three T-cell locomotion measures (i.e., the translational speed, turning angle speed, and meandering index), the following numerical simulation protocol was used, which is close to the original experimental protocol (22). First, the same 2D 412 ×412 μm^2^ domain was used, in which we initialized 4,489 squarely tiled T cells with 3-μm radii and η ≈ 80% packing density. The initial direction of the intrinsic motility force was generated randomly for all cells. The positions of cells were saved every 30 s during 10 numerical experiments of 30-min simulation time after a 30-min pre-run to randomly mix the cells. Cells with total displacements <27 μm were excluded as was done in the original experimental protocol. Likewise, cells that passed through the boundary and left the imaging volume were also excluded. The saved cell positions were post-processed to calculate the target metrics (defined in Figure 1D), which were pooled together to form three separate distributions. The pooled cell motility distributions were calibrated with *in vivo* data. The simultaneous adjustment of all distributions was computationally challenging due to the different uncorrelated aspects of cell migration captured in each of the motility metric as previously outlined (22). Figure 1G shows the best-fit cumulative distribution functions (CDFs) of the calibrated model with the baseline parameter set from Table 1 and the target experimentally observed distributions with Kolmogorov–Smirnov statistics and *p*-values describing the discrepancy between CDFs.

The evolution of the above multicellular system was simulated over a 12-h period. The visualization of the systems spatiotemporal dynamics is presented in Movie S1. Figures 2B,C shows the kinetics of median velocities of antigen-specific CD4^+^ T and CD8^+^ T cells, and the median distances between the T cells and the centroid of their cognate antigen-presenting DCs throughout 12 h of an *in silico* experiment. The model demonstrates that antigen-specific CD8^+^ T cells that interact with their cognate CD8α^int^ DCs but not the CD4^+^ T cells decrease their velocities, move closer to the area of DCs in the first 4–6 h, and remain there with low velocities afterward. Figures 2B,C is quantitatively consistent with experimental data shown in Figures 1E,F, and in Figure 2B from Kitano et al. (24).

### Quantitation of the DC and T-Cell Contact Interactions

**Figure 3:**
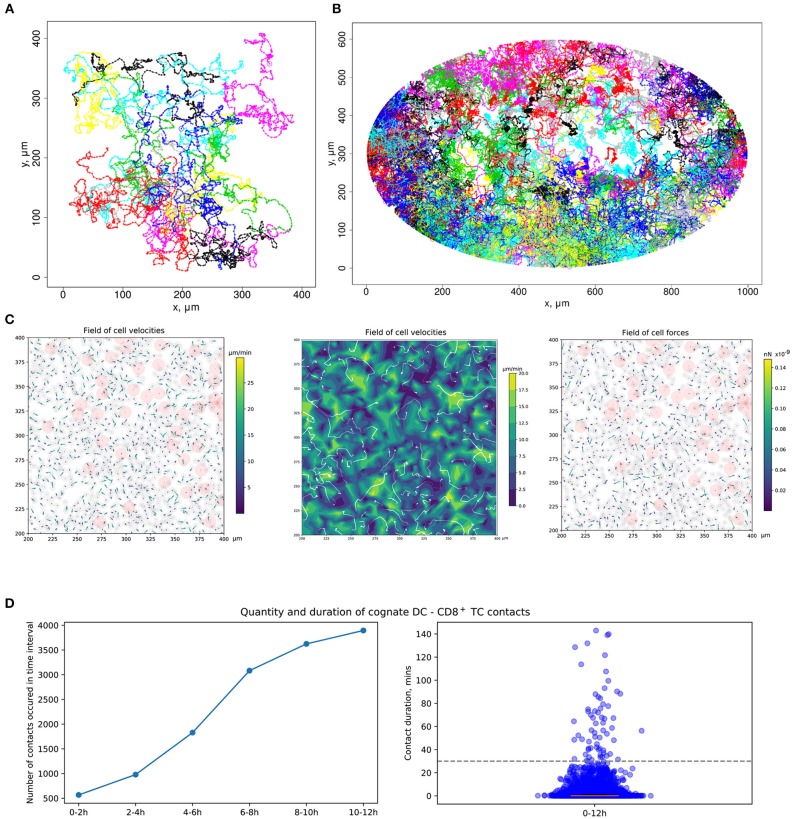
Quantitation of immune cell motility, driving forces, and contacts. **(A)** Representative example of individual cell trajectories obtained with numerical realization of the calibrated model. The trajectories illustrate the 5-h dynamics of 15 cells randomly chosen from 4,489 cells presented in the 412 ×412 μm^2^ domain with periodic boundary conditions. **(B)** Twelve-hour multicellular dynamics of T-cell trajectories in a lymph node obtained by numerical simulation with an initial configuration specified in Figure 2A. Only cells with total displacement longer than 27 μm are shown. **(C)** Values of forces and cell velocities driving the multicellular system dynamics in a square subdomain of a LN. In a center pane, the velocity field is represented as a contour plot of the field of cell velocity magnitudes linearly interpolated at uniform grid, as well as detected streamlines of possible cell flow patterns. **(D)** Kinetics of the numbers of cognate DC–T cell contacts at different stages of the simulation and distribution of durations of all cognate contact durations occurring within a 12-h simulation. DC, dendritic cell.

The calibrated mathematical model of T-cell locomotion was validated by confronting its predictions with data from the intranodal spatiotemporal dynamics of different immune cell subsets after soluble antigen immunization presented in a recent experimental study (24). The data specify the evolution of the distances between the centroid of the migratory DC area and individual CD4^+^ T and CD8^+^ T cells. The model was adjusted to the functional configuration of skin-draining LNs specified in the above study. A representative example of the numerical simulation of individual cell trajectories is shown in Figure 3A. An example of multicellular dynamics in a LN during 12 h is shown in Figure 3B.

#### Quantitation of the Forces Determining T-Cell and DC Motility and Their Interaction

To consider DCs in multicellular system simulations, we carried out a parameterization of their intrinsic motility forces and the intercellular forces for contacts between (1) two DCs, (2) antigen-presenting DCs and antigen-specific T cells, and (3) antigen-presenting DCs and polyclonal T cells. The values of the corresponding parameters are presented in Table 2. The physical forces driving the dynamics of individual cells in the LN and the respective velocities of the cells predicted by the model are shown in Figure 3C. We assume that the intrinsic motility of DCs can be represented by the same type of force function as that for T cells (Figure 1C); however, due to their much smaller average velocity, the respective DC force function value was of small magnitude. The adhesive force for cognate contacts (i.e., of antigen-specific T cells with antigen-presenting DC) is around 100 times higher (~1 nN) than the non-specific adhesion force for T cell/T cell contacts (36). We also implemented a computational procedure to temporarily arrest the motility for T cells in a sufficiently long cognate contact (see section Materials and Methods).

#### Ag-Specific CD8^+^ T Cells Migrate Toward Cross-Presenting DCs and Form Cognate Contacts With Them

Figure 3D presents the model prediction for the kinetics of the number of cognate DC–CD8^+^ T-cell contacts occurring at different time intervals during the *in silico* simulation. Antigen-specific CD8^+^ T cells robustly increase the number of contacts with DCs over time in the process of T-cell zone scanning for antigen-presenting target cells. Although most of the cognate contacts are of short duration, i.e., they last for <5 min, the distribution has a heavy tail of stable more than 1-h length contacts. These predictions are in agreement with previous data (45).

### CTL Frequency Needed to Locate HIV-Infected Target Cells Before Viral Release

During viral infections, the induction of cellular immune responses takes place in secondary lymphoid organs such as LNs and spleen. Antigen-presenting cells such as DCs take up antigens and migrate to LNs to encounter specific lymphocytes, e.g., CD4^+^ T and CD8^+^ T cells, to induce their activation and differentiation into effector and memory cell subtypes (46). The low frequency of antigen-specific T cells in unprimed hosts turns the scanning of cognate DCs by specific T cells in a highly crowded cellular LN environment into a “needle-in-a-haystack” problem (47). It was revealed that optimal LN scanning depends on a combination of intrinsic T-cell motility, the chemokine milieu, and the microarchitecture of the LN (1). When virus-infected DCs reach the LN, the less the time needed to locate virus-specific T cells and to form stable DC–T cell contacts, the more likely is that the precursor CTL activation will happen before the viruses will be released from infected cells, therefore making the elimination of local clusters of infection spread more probable. This aspect of CD8^+^ T-cell activity is crucial for a prompt activation of specific CTL immune responses and the elimination of viruses. The precursor frequency in blood can be as small as 0.0001% (48), reaching about 5–10% in the chronic stage of an HIV infection (49). The here-developed physics-based model of T-cell dynamics can be directly used to study the efficiency of scanning the paracortical T-cell zone of the LN for target cells expressing cognate antigen as a function of the frequency of CTL and their motility.

Development of an effective AIDS vaccine remains a global priority, and there is a need for a vaccine to induce cellular immune responses capable of eradicating or efficiently containing virus replication (50). Experimental studies with attenuated SIV vaccines indicated that SIV-specific CTLs, if present in sufficient frequencies, can completely control and even clear an infection (19). Similar to SIV, HIV infection is sustained by the activation of CD4^+^ T cells, which occurs in the form of transient bursts in the local microenvironment of lymphoid tissues (51, 52). The proximal activation and transmission involving latently infected cells represent locally propagating events (53). Therefore, we applied our calibrated model of spatial immune cell dynamics in LNs to study the necessary conditions for effector HIV-specific CTLs to promptly locate HIV-infected target cells before they can release viral progeny. We consider only one HIV-infected cell in the computational domain, which is consistent with the frequency of productively infected CD4^+^ T cells of about 0.0001–0.001 (54). Specifically, the newly infected target cell should be located by the nearby effector cells before it can release viral progeny, i.e., before completion of the 18–24 h life cycle of HIV (20).

**Figure 4:**
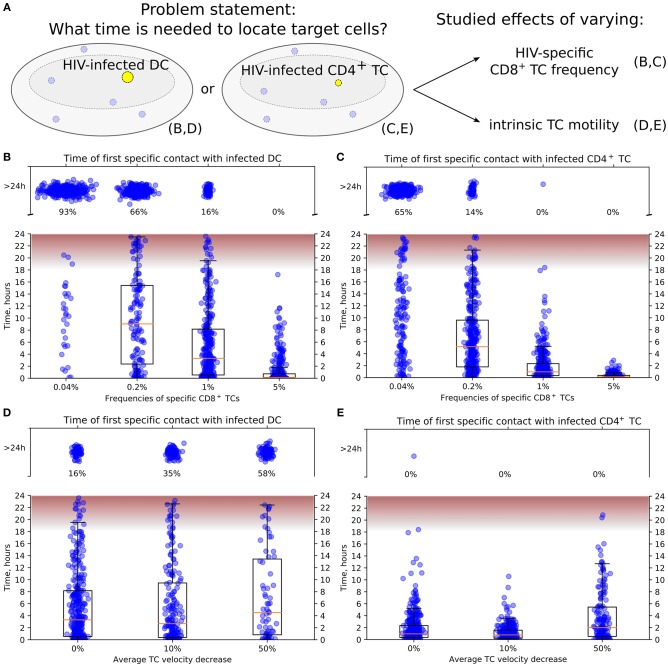
Conditions to locate HIV-infected target cells within a LN before viral release. **(A)** General scheme of *in silico* simulations. Time since the HIV-infected target cell was introduced until it was located by effector HIV-specific CTLs was measured in 24-h simulations. The infected cell was either non-motile DC **(B,D)** or motile CD4^+^ T cell **(C,E)**. In **(B,C)**, the precursor frequency, i.e., the frequency of effector T cells, was varied, from 0.04 to 5%. In **(D,E)**, the effect of decreased intrinsic motility of T cells was studied. The average T-cell velocity was decreased up to 50%. In all plots, the fraction of cases with location time >24 h is indicated, thus providing the estimates for probability to locate target cells within 24 h. The time range between the start and the peak HIV release from infected T cells (20) is shown in pink. It is used to estimate the probability of a virus burst to escape effector CTLs and, thus, to contribute to the spread of HIV-infected cells within a LN. TC, T cell; DC, dendritic cell.

The overall simulation setup is the same as described in the model validation subsection above. Randomly chosen cells in the stochastically generated multicellular system configurations representing the LN cortex zone were marked as infected in yellow (see Figure 4A). Both the motile CD4^+^ T cells and the non-motile DCs were considered as HIV-infected targets. In simulations, we varied the frequency of HIV-specific CD8^+^ T cells and the intrinsic motility of T cells (searching CD8^+^ T cells, infected- and uninfected CD4^+^ T cells) (Figure 4A) to analyze the effect of variations on the target cell detection time. A 10-fold range of HIV-specific CD8^+^ T-cell frequencies typical for HIV infection, i.e., from 0.4 to 5%, was examined. The intrinsic motility of T cell was varied within 100 and 50% relative to the calibrated baseline parameters of average T-cell velocity (see details in the Supplementary Text). A decreased intranodal T-cell motility (of searching CD8^+^ T cells, infected- and uninfected CD4^+^ T cells) is expected to take place during the chronic stage of an HIV infection when LN tissues become fibrotic, i.e., when collagen formation in T-cell zones takes place (55). Then, T cells have to move through increased collagen deposition with major consequences for search patterns (56). In addition, CD4^+^ T-cell migration is also inhibited by the HIV-1 Nef protein as shown in chemotaxis assays (57). In our study, the motility of all considered types of T-cell subsets, i.e., the searching CD8^+^ T cells, uninfected CD4^+^ T cells, and infected CD4^+^ T cells, is decreased uniformly.

Figures 4B,C illustrate the model predictions for the decrease of time to locate HIV-infected target cells with the increase of HIV-specific CD8^+^ T-cell frequency. The modeling results imply that 5% is a sufficient effector CTL frequency for a timely detection of both types of target cells within 18 h post-infection, i.e., before the beginning of HIV release from productively infected cells (20). A stepwise increase by five-fold of the HIV-specific CTL frequency from 0.04 to 5% increases the probability of detection of HIV-infected cell within 24 h from 0.07 to 0.34 to 0.84 and to 1, respectively. In addition, the model shows that infected motile CD4^+^ T cells are located faster than non-motile DCs with the probability of detecting them within 24 h increasing from 0.35 to 0.86 and to 1 with a CTL frequency rising from 0.04 to 0.2% and to 1%, respectively.

Figure 4D shows the increase of time to locate infected non-motile DCs for a decrease of the T-cell motility from a basal level by 10 and 50% considering an HIV-specific CD8^+^ T-cell frequency of 1%. If the average T-cell velocity is decreased by 50%, then the probability to locate DCs within 24 h is <0.5. Figure 4E depicts a similar dynamics for locating motile infected CD4^+^ T cells. Note that the motile targets were located within 24 h even with a 50% decrease of the average CD8^+^ T-cell velocity in all performed simulations.

## Discussion

We have developed a biophysics-based computational model of T-lymphocyte motility that is calibrated using empirical *in vivo* data on T-cell migration in LN tissue representing three spatial metrics of multicellular systems behavior, i.e., translational speed, turning angle speeds, and meandering index. The model provides the tool to quantify the velocity and the driving force fields in the LN. It enabled us to predict frequency and motility parameters that are required for a timely detection of productively HIV-infected cells within LNs before they release viral progeny. As such, our study provides a quantitative guide for an informed design of HIV vaccines. Furthermore, as the immunological principles of antigen-specific T-cell activation and immune surveillance imbedded in our model also apply to other infections and cancers, our findings may be used to define the general requirements for any efficient immunotherapeutic intervention against pathogens or cancers in relation to disease-specific parameters and states of lymphoid tissue and T cells. Thus, our model has a significant potential to guide the search for better and more efficient immunotherapies in the near future.

Other processes, e.g., chemotaxis, haptotaxis, and others, can influence the efficacy of target cell search by CTL. The impact of chemotactic migration of T cells toward DCs has been computationally analyzed using a cellular Potts Model (58), an agent-based model (59), and a multicompartmental spatially resolved stochastic model of T-cell circulation (60). The results suggest that the chemoattraction toward target cells modestly speeds up the search process for T cells that successfully find the chemokine-producing DCs. However, a qualitative model presented in (59) suggested that with even weak chemotaxis, substantially lower numbers of CTL are required for sterilizing immunity. Further data-based model-driven research is needed to clarify the contribution of chemotaxis to T-cell migration under normal conditions and during inflammation (61).

Phenomenological Ordinary Differential Equation (ODE) models may also be developed to simulate the interactions between cell populations in the LN. However, these models are not suitable for the present study for three reasons. First, data on T-cell motility in the LN cannot be directly used to calibrate such models, thereby limiting the validity of their predictions. Second, the objective of our study, which is the early detection of HIV-infected T cells and DCs, requires the monitoring of the spatial density of T cells in the LN rather than the total number of T cells. Changes in the spatial distribution of T cells in the LN can be related to spatial mechanisms such as chemotaxis and migration. Therefore, it is crucial to consider spatial aspects in the model. Finally, ODE models based on “mass action”- or “predator–prey”-type parameterizations would require the parameter values specifying a per capita killing rate of target cells. The respective parameter can be determined by the mean time needed for a migrating CTL to locate infected cells. *A priori* estimates of this parameter are not available. It is the spatially resolved model-based simulation that needs to be implemented in order to quantify the killing rate coefficient of the ODE model.

Moving from phenomenological models of spatiotemporal dynamics of immune processes (e.g., the compartmental models, CAMs) to a physics-based description of immune cell migration in complex multicellular tissue environments presents a challenge to mathematical immunology. Advances in the direct visualization of antigen-specific T-cell mobility during their search for and their interaction with antigen-presenting cells within LNs set the basis for diverse modeling approaches (7, 10, 11), which have been so far based on *ad hoc* postulated rules of cell behaviors. Our study gives a biophysics perspective on coordinated cell motility in lymphoid tissues, thus extending the range of modeling tools available for implementing integrative approaches to the exploration of the immune system.

CPMs have also been applied previously to study intranodal T-cell migration (58). The CPM framework is a valuable tool for a phenomenological description of multicellular patterning, providing realistic simulations of morphological changes for various cell types. The strength of this approach stems from its flexible energetic formalism that allows for extensions to incorporate various biological processes (62). Although the CPM framework has a richer potential for describing individual cell dynamics, including the cell shape, this comes at the expense of (i) a higher-dimensional representation of the cell configuration (e.g., the number of voxels or pixels), (ii) the use of phenomenologically rather than biophysically defined parameters, and (iii) a much higher computational cost to perform simulations required to explore T-cell search strategies. Besides, there is no direct correspondence of most of the CPM parameters with biophysical properties of cells, and the meaning of some CPM parameters is still under debate (62, 63). Moreover, CPM temporal kinetics obtained with the modified Metropolis algorithm does not preserve the detailed balance condition for the underlying stochastic process. This implies that the exact relation between forces of cell interactions and energy terms of CPMs cannot be obtained even for the overdamped dynamics approximation (62, 63).

Computational modeling of multicellular dynamics in lymphoid tissues provides a theoretical tool to be used for a better understanding of the determinants of efficient immune responses against pathogens with a final aim of an optimal manipulation of the immune systems performance (2, 15). Given that the quest for an effective HIV vaccine remains a global priority (64) and that the localization, migration, and frequency of CTLs in LNs determine the extent of virus elimination (17, 19, 36, 56, 65), we sought to use our modeling approach to define threshold frequencies of CTLs in LNs for protection against HIV. Since an HIV infection can influence (i) CD4^+^ T-cell motility by a direct mechanism involving the HIV Nef protein and (ii) CTL locomotion via an indirect mechanism related to the induction of lymphatic tissue fibrosis, we considered both phenomena to predict the effect of reduction of T-cell motility. We estimated that the frequency of antigen-specific CTL should be about 5% to timely detect and completely eliminate productively infected DCs within 18 h. The time reduces to 4 h for productively infected CD4^+^ T cells, which are motile. For an HIV-specific T-cell frequency of 1%, we computed that the inhibition of CTL locomotion by two-fold would reduce the probability of detection of infected target cells within 24 h post-infection from 0.84 to 0.42. Thus, the requirements for a prophylactic vaccine for seronegative individuals and an immunotherapeutic intervention of already HIV-infected individuals may differ significantly and are influenced by the state of the lymphatic tissue structure.

Understanding the spatiotemporal dynamics of immune cells globally in the lymphatic system and locally in LNs is considered to be a prerequisite for the development of novel immune interventions in the context of HIV cure strategies (15, 56). To this end, mathematical tools are being increasingly applied to predict the impacts of trafficking and motility parameters on the efficiency of immune surveillance in health and disease. For example, an optimal surveillance strategy for T cells was analyzed by compartmental modeling of their systemic recirculation and LN transit times using a multicompartmental consideration (66). The protective effect of increased CD4^+^ T-cell trafficking on the dynamics of HIV infection has been recently shown using another compartmental model (67), thus providing a basis for considering cell trafficking as an adjunct therapy option. A multiscale model of *Mycobacterium tuberculosis* infection including an agent-based description of the cellular movement in a two-dimensional simulation grid representing the granuloma was developed and calibrated using non-human primates to derive the prediction of parameters underlying granuloma sterilization (8). However, such modeling attempts are still rare.

In conclusion, the large number of existing mathematical models based on low-resolution descriptions of immune functions has to be further extended and embedded into physiologically distinct compartments and 3D morphological constraints inherent to cells, tissues, and the whole organism. This will then allow the research community not only to get a better quantitative understanding of immune system functioning in infections such as HIV but also enable to build integrative models for antiviral and immunomodulatory drugs of various physical and chemical nature as well as the effects of adoptive cell transfer therapies. We believe that a comprehensive approach to combination therapies based on ART and immunomodulatory drugs affecting a range of processes, including LN fibrosis, the exhaustion of CTLs, and T-cell motility, should rely on formulation and implementation of hybrid spatially resolved multiscale mathematical models of virus infections (8, 9, 68). The here-developed model offers a broadly applicable generic mathematical tool for linking individual and coordinated cell behaviors that can be used for *in silico* studies to embed immune processes into their spatial context. The physics-based computational model of multicellular dynamics of the immune response in lymphoid tissues provides a solid module that can be universally used in systems immunology studies (2, 6) for the benefit of patients suffering from chronic virus diseases.

## Author Contributions

DG, AM, VV, and GB conceived and designed the study. DG, VV, AM, and GB wrote the paper. DG, AB, and NB performed the computational implementation of the model. DG performed the model calibration.

### Conflict of Interest Statement

The authors declare that the research was conducted in the absence of any commercial or financial relationships that could be construed as a potential conflict of interest.
