# Corrigendum: Spatial Lymphocyte Dynamics in Lymph Nodes Predicts the Cytotoxic T Cell Frequency Needed for HIV Infection Control

**DOI:** 10.3389/fimmu.2019.01538

**Published:** 2019-07-03

**Authors:** Dmitry Grebennikov, Anass Bouchnita, Vitaly Volpert, Nikolay Bessonov, Andreas Meyerhans, Gennady Bocharov

**Affiliations:** ^1^Moscow Institute of Physics and Technology, National Research University, Dolgoprudny, Russia; ^2^Marchuk Institute of Numerical Mathematics, Russian Academy of Sciences, Moscow, Russia; ^3^Peoples' Friendship University of Russia (RUDN University), Moscow, Russia; ^4^Division of Scientific Computing, Department of Information Technology, Uppsala University, Uppsala, Sweden; ^5^Institut Camille Jordan, UMR 5208 CNRS, University Lyon 1, Villeurbanne, France; ^6^INRIA Team Dracula, INRIA Lyon La Doua, Villeurbanne, France; ^7^Institute of Problems of Mechanical Engineering, Russian Academy of Sciences, Saint Petersburg, Russia; ^8^Infection Biology Laboratory, Department of Experimental and Health Sciences, Universitat Pompeu Fabra, Barcelona, Spain; ^9^Institució Catalana de Recerca i Estudis Avançats (ICREA), Barcelona, Spain; ^10^Sechenov First Moscow State Medical University, Moscow, Russia

**Keywords:** lymphoid tissue, cell motility, HIV infection, cytotoxic T cell scanning, multicellular dynamics, dissipative particle dynamics, stochastic differential equation

In the original article, there was a typo in Figure 2A color legend as published. The colored circles denoting Ag-specific and non-specific T cells should be swaped. That is, the dark green color should represent Ag-specific CD4^+^ TCs, the light green color—non-specific CD4^+^ TCs; the dark blue color should represent Ag-specific CD8^+^ TCs, the light blue color—non-specific CD8^+^ TCs. The corrected Figure 2 appears below.

**Figure 2:**
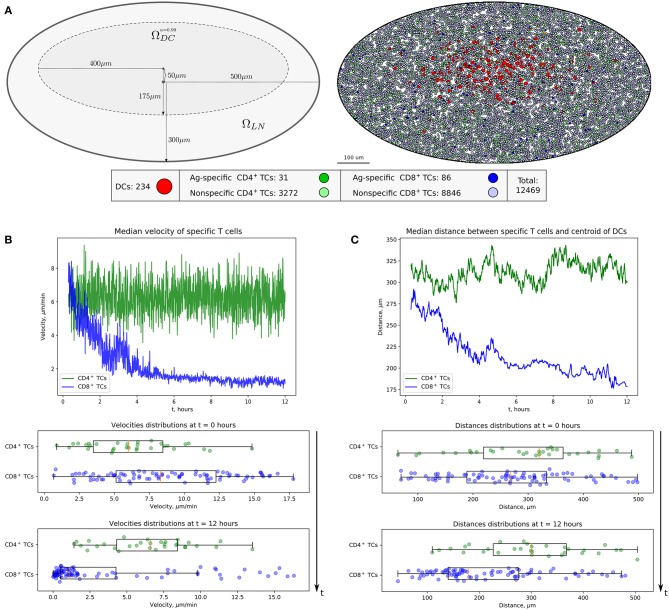
Heterogeneous dynamics of T cells in LNs. **(A)** The scheme of a LN and illustration of the initial configuration generated for simulations. DCs, CD4^+^ T cells, and CD8^+^ T cells are placed within a LN as described in the Supplementary Text with total cellularity of 12,469 cells, ≈80% packing density and ≈1% precursor frequency. **(B)** Twelve-hour kinetics of median velocities of antigen-specific CD8^+^ T and CD4^+^ T cells, and their distributions at the start and at the end of a 12-h simulation. **(C)** Twelve-hour kinetics of median distances from T cells to the centroid of DCs, measured for antigen-specific CD8^+^ T and CD4^+^ T cells, and their distributions at the start and at the end of a 12-h simulation. TC, T cell; DC, dendritic cell.

The authors apologize for this error and state that this does not change the scientific conclusions of the article in any way. The original article has been updated.

