# Supplementary material for: Spatial Lymphocyte Dynamics in Lymph Nodes Predicts the Cytotoxic T Cell Frequency Needed for HIV Infection Control
Source: Front Immunol. 2019 Jun 11;10:1213. doi: 10.3389/fimmu.2019.01213 (PMC6579925; doi:10.3389/fimmu.2019.01213)
Supplement: Supplementary file 2 [file Data_Sheet_1.pdf]

Spatial Lymphocyte Dynamics in Lymph Nodes Predicts the Cytotoxic T cell Frequency Needed for HIV Infection Control

Dmitry Grebennikov[1,2,3*], Anass Bouchnita[4], Vitaly Volpert[3,5,6], Nikolay Bessonov[7], Andreas Meyerhans[8,9], Gennady Bocharov[2,10*]

[1]Moscow Institute of Physics and Technology (National Research University), Dolgoprudny, Moscow Region, Russia

[2]Marchuk Institute of Numerical Mathematics, Russian Academy of Sciences, Moscow, Russia

[3]Peoples' Friendship University of Russia (RUDN University), Moscow, Russia

[4]Division of Scientific Computing, Department of Information Technology, Uppsala University, Uppsala, Sweden

[5]Institut Camille Jordan, UMR 5208 CNRS, University Lyon 1, Villeurbanne, France

[6]INRIA Team Dracula, INRIA Lyon La Doua, Villeurbanne, France

[7]Institute of Problems of Mechanical Engineering, Russian Academy of Sciences, Saint Petersburg, Russia

[8]Infection Biology Laboratory, Department of Experimental and Health Sciences, Universitat Pompeu Fabra, Barcelona, Spain

[9]Institució Catalana de Recerca i Estudis Avançats (ICREA), Barcelona, Spain

[10]Sechenov First Moscow State Medical University, Moscow, Russia

*** Correspondence:**

Dmitry Grebennikov, dmitry.ew@gmail.com

Gennady Bocharov, bocharov@m.inm.ras.ru

Supplementary Material

Supplementary Text

We estimated the numbers for immune cell subsets, i.e. DCs, $CD4^+$ T cells and $CD8^+$ T cells, in lymph nodes based on the data from (Kitano et al., 2016). First, the numbers of DCs were estimated by rescaling the whole LN flow cytometry counts to a computational domain. We assume that flow cytometry reveals about 10% of the total cellularity (Mario Novkovic, personal communication) and that the computational domain represents a central section of a bean-shaped LN of 10 μm depth. The semi-axes of the spheroid LN representation are $a = \frac{1}{2}1000\mu m$, $c = \frac{1}{2}600\mu m$, leading to the following values of the volumes of a LN: $V_{\mathrm{LN}} = \frac{4\pi}{3}a^2c \approx 0.3 \times 10^9 \mu m^3$, and the slice: $V_{\mathrm{Slice}} = \pi \mathrm{ac} \cdot z_{\mathrm{depth}} \approx 4.7 \times 10^6 \mu m^3$. We note that rescaling the cell counts from (Kitano et al., 2016) using the assumptions stated above resulted in a biologically realistic dense packing of the computational domain ($\eta \approx 80\%$).

Given that the total protein mass per APC is in the range between 64 ± 14 pg and 95 ± 25 pg (for different dendritic cell subtypes) (Wiśniewski et al., 2014), and the estimation that the fraction of dry solids in a cell constitutes about 27% of the total mass (Brown, 1991, Illmer et al., 1999), we set the mass of wet APC to be 350 pg. The same approach was used to define the T cell mass. To estimate the T cell mass, we used lymphocyte wet weight measurements from (Segel et al., 1981). The here chosen value of 215 pg corresponds to a protein mass of 60 pg, which is in the range of the total protein mass of $CD4^+$ T cells (Wiśniewski et al., 2014). As the protein mass of $CD8^+$ T cells is on average 1.35 times larger than the protein mass of $CD4^+$ T cells (Wiśniewski et al., 2014), we set the mass of wet $CD8^+$ T cells to be 290 pg.

The T cell radius was set to 3 μm (Turgeon, 2005). APC diameter estimates vary from 7 to 15 μm (Wiśniewski et al., 2014; Goya et al., 2008). Most likely, the range is so wide because of different dendritic cell subtypes analyzed in references, and also because a dendritic cell represents a spherical body with numerous dendrites around. In our model, we define APCs as spherical circles with 13 μm diameter, which incorporates the area of dendrites, whereas we change the range of adhesive area in repulsion force to lie in (0.5r, r) ($\lambda_{DC} = 0.5$). Thus, the actual repulsive body of an APC is 6.5 μm, and the outer part $x \in$ (6.5, 13) μm is an adhesive dendritic area (see Figure 1C).

The adhesive strength between T cells, which is a weak nonspecific electrical force (Bell, 1978), is of the order of 0.01 to 0.03 nN (Basu and Huse, 2017, Kong et al., 2009). It was set to 0.01 nN. The specific adhesive strength between T cells and APCs is estimated to be around 1 nN, based on single cell force spectroscopy measurements (Lim et al., 2012). Also, from that publication, we roughly estimate the viscous

damping coefficient: the maximal force measured on the cantilever with a T cell slowly approaching an APC was 1 nN, which leads to $\mu \approx 0.1\ nN \cdot min\ /\mu m$ if divided by the typical lymphocyte velocity of 10 $\mu m/min$ (Miller et al., 2003). We tuned this parameter to $\mu = 0.2 nN \cdot min/\mu m = 12\, g/s$ during calibration. The adhesive strength between APCs is of the same value as the nonspecific adhesive strength between T cells.

We set the motility magnitude to be sampled from N(3, 0.3) nN distribution, so that percentiles $P_1 \approx 2.3$ nN, $P_{99} \approx 3.7$ nN are positive and below the mechanical force of ~ 5 nN, which is exerted by cytotoxic cells on target cells to potentiate their killing by destructing their membrane (Basu et al., 2016). The mean and standard deviation were also manually calibrated to ensure that the maximal cell velocity is <25μm/min and that their overall profile matches a target cumulative distribution.

The standard deviation for the motility turning angle $\sigma(\alpha_{TC})$, the coefficient c_{inh} that takes into account the level of CIL influence and the coefficient β parameterizing a negative correlation between the motility magnitude and the turning angle, were set according to the models they originate from (Read et al., 2016, Zimmermann et al., 2016), and were tuned to match the T cell motility profile.

When performing *in silico* simulations to study the effect of decreased T cell motility on target cell location efficiency (Figures 4D and 4E), the parameter η_i was used to decrease the intrinsic motility. We evaluated that a 10% decrease of the average T cell velocity from the baseline value corresponds to $\eta_i = 0.75$, the 50% decrease corresponds to $\eta_i = 0.33$. To obtain statistics for Figure 4, N=400 realizations were computed for each scenario of *in silico* simulations.

Visualization of the spatiotemporal dynamics of the multicellular system simulated over a 3-hour period is presented in *Movie S1*.

Supplementary references:

1. Kitano M, Yamazaki C, Takumi A, Ikeno T, Hemmi H, Takahashi N, et al. Imaging of the cross-presenting dendritic cell subsets in the skin-draining lymph node. *Proc. Natl. Acad. Sci.* (2016) 113:1044–1049. doi: 10.1073/pnas.1513607113.

2. Wiśniewski JR, Hein MY, Cox J, Mann M. A “proteomic ruler” for protein copy number and concentration estimation without spike-in standards. *Mol. Cell Proteomics. MCP* (2014) 13:3497–3506. doi: 10.1074/mcp.M113.037309.

3. Brown GC. Total cell protein concentration as an evolutionary constraint on the metabolic control distribution in cells. *J. Theor Biol.* (1991) 153:195–203. doi: 10.1016/S0022-5193(05)80422-9

4. Illmer P, Erlebach C, Schinner F. A practicable and accurate method to differentiate between intra- and extracellular water of microbial cells. *FEMS Microbiol. Lett.* (1999) 178:135–139.

5. Segel GB, Cokelet GR, Lichtman MA. The measurement of lymphocyte volume: importance of reference particle deformability and counting solution tonicity. *Blood* (1981) 57:894–899.

6. Turgeon ML. *Clinical hematology: theory and procedures*. 4th ed. Philadelphia: Lippincott Williams & Wilkins (2005).

7. Goya GF, Marcos-Campos I, Fernández-Pacheco R, Sáez B, Godino J, Asín L, et al. Dendritic cell uptake of iron-based magnetic nanoparticles. *Cell. Biol Int.* (2008) 32:1001–1005. doi: 10.1016/j.cellbi.2008.04.001.

8. Bell GI. Models for the specific adhesion of cells to cells. *Science* (1978) 200:618–627. doi: 10.1126/science.347575.

9. Basu R, Huse M. Mechanical communication at the immunological synapse. *Trends Cell. Biol.* (2017) 27:241–254. doi: 10.1016/j.tcb.2016.10.005.

10. Kong F, García AJ, Mould AP, Humphries MJ, Zhu C. Demonstration of catch bonds between an integrin and its ligand. *J. Cell Biol.* (2009) 185:1275–1284. doi: 10.1083/jcb.200810002.

11. Lim TS, Goh JKH, Mortellaro A, Lim CT, Hämmerling GJ, Ricciardi-Castagnoli P. CD80 and CD86 differentially regulate mechanical interactions of T-cells with antigen-presenting dendritic cells and B-cells. *PLOS ONE* (2012) 7:e45185. doi: 10.1371/journal.pone.0045185.

12. Miller MJ, Wei SH, Cahalan MD, Parker I. Autonomous T cell trafficking examined *in vivo* with intravital two-photon microscopy. *Proc. Natl. Acad. Sci. USA* (2003) 100:2604–2609. doi: 10.1073/pnas.2628040100.

13. Basu R, Whitlock BM, Husson J, Le Floc'h A, Jin W, Oyler-Yaniv A, et al. Cytotoxic T cells use mechanical force to potentiate target cell killing. *Cell* (2016) 165:100–110. doi: 10.1016/j.cell.2016.01.021.

14. Read MN, Bailey J, Timmis J, Chtanova T. Leukocyte motility models assessed through simulation and multi-objective optimization-based model selection. *PLOS Comput. Biol.* (2016) 12:e1005082. doi: 10.1371/journal.pcbi.1005082.

15. Zimmermann J, Camley BA, Rappel W-J, Levine H. Contact inhibition of locomotion determines cell–cell and cell–substrate forces in tissues. *Proc. Natl. Acad. Sci. USA* (2016) 113:2660–2665. doi: 10.1073/pnas.1522330113.

Supplementary Movies

Legend for supporting video file "Movie_S1"

Visualization of the spatiotemporal dynamics of the multicellular system simulated over a 50 minutes period. The following cell subsets are considered: CD4$^+$ T cells, CD8$^+$ T cells and cross-presenting migratory CD8$^{\alpha int}$CD103^{hi} DCs. DCs, CD4$^+$ T cells and CD8$^+$ T cells are placed within a total cellularity of 12469 cells, 80% packing density and 1% precursor frequency. Both T cell subsets are distributed uniformly through the whole LN, while migratory DCs are found mainly deep in the paracortex area. The spatial positions for DC locations are iteratively sampled from the 2D Gaussian distribution with a 99-percentile ellipse DC and accepted if the DC with sampled coordinates lies within the LN domain and does not overlap with the other seeded DCs.

Supplementary Figures

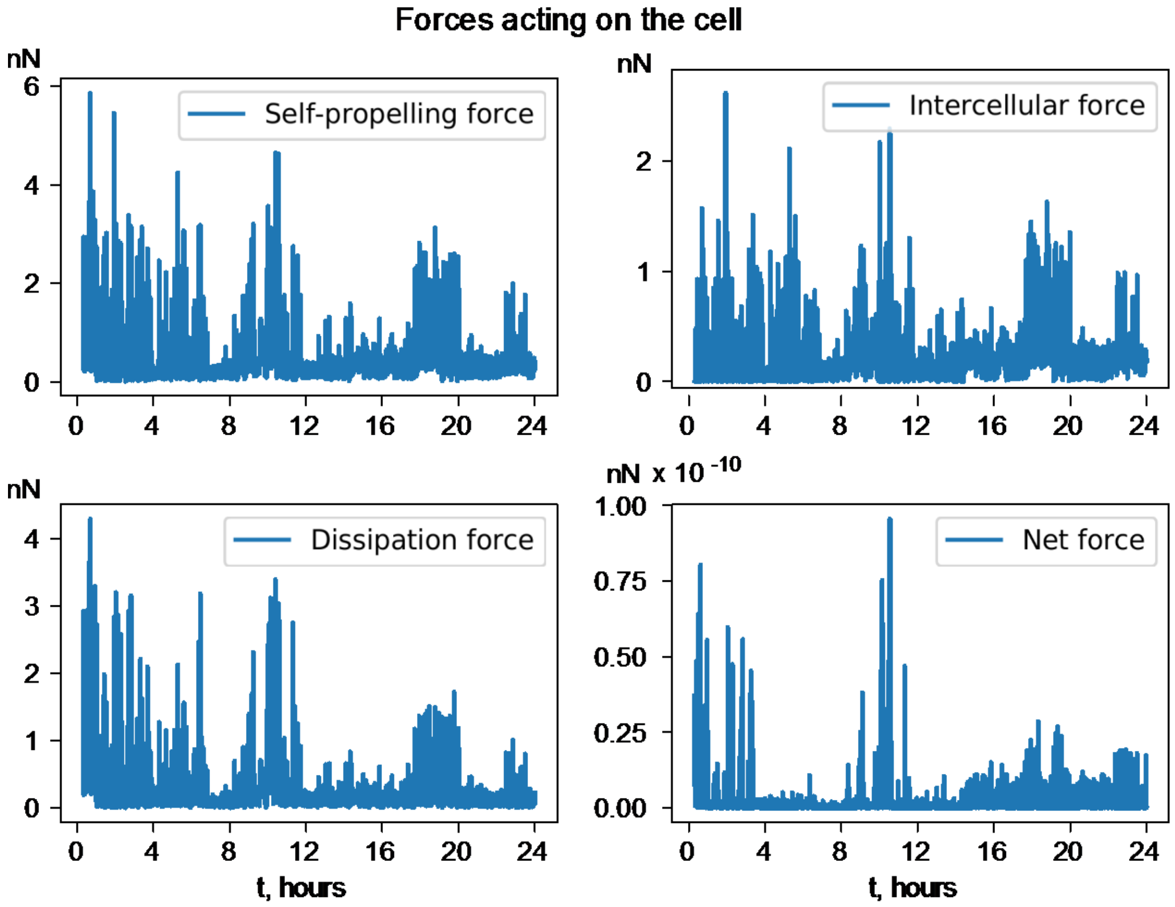

Figure S1. The absolute values of the components of the net force acting on the randomly selected CD8$^+$ T cell in 24-hour of *in silico* simulations.
